# Comparative performance of invasive, semi-invasive, and non-invasive insulin resistance-related indices across prediabetes and normoglycemia

**DOI:** 10.1371/journal.pone.0351950

**Published:** 2026-06-26

**Authors:** Gitika Bhasin, Rucha S. Dafale, Shobha U. Kamath, Mukhyaprana M. Prabhu, Suman Rawat, Mohit Kumar, Annapoorna K, Sahana Shetty, Lavya Shetty, Vasanthalaxmi K, Akhilesh K. Pandey, Manjula S. D

**Affiliations:** 1 Division of Yoga, Centre for Integrative Medicine and Research, Manipal Academy of Higher Education, Manipal, Karnataka, India; 2 Department of Physiology, Kasturba Medical College, Manipal Academy of Higher Education, Manipal, Karnataka, India; 3 Department of Biochemistry, Kasturba Medical College, Manipal Academy of Higher Education, Manipal, Karnataka, India; 4 Department of Medicine, Kasturba Medical College, Manipal Academy of Higher Education, Manipal, Karnataka, India; 5 Department of Wellness and Yogic Science, School of Health Sciences and Technology, Dr. Vishwanath Karad MIT-World Peace University, Pune, Maharashtra, India; 6 Kasturba Medical College, Manipal Academy of Higher Education, Manipal, Karnataka, India; 7 Department of Endocrinology, Kasturba Medical College, Manipal Academy of Higher Education, Manipal, Karnataka, India; 8 Department of Community Medicine, Kasturba Medical College, Manipal Academy of Higher Education, Manipal, Karnataka, India; The Chinese University of Hong Kong, HONG KONG

## Abstract

Insulin resistance, a defining abnormality in the development of type 2 diabetes, often emerges silently during prediabetes. This study evaluated the comparative performance of multiple insulin resistance-related indices across adults with prediabetes and normoglycemia. A cross-sectional analysis was conducted among 100 adults aged 25–55 years, equally divided between the two glycemic groups. Twenty-five candidate indices were examined, spanning blood-based indices, lipid–anthropometry combinations, and non-invasive anthropometric and body-composition measures, including several newly derived indices. Correlation analysis was performed to assess the relationship between candidate indices and the Homeostasis Model Assessment for Insulin Resistance, followed by logistic regression and receiver operating characteristic curve evaluation for indices showing significant correlations. Individuals with prediabetes demonstrated significantly higher adiposity and differences in metabolic parameters compared to individuals with normoglycemia (p < 0.05 for most variables). Seventeen of the twenty-five candidate indices showed significant correlations with the Homeostasis Model Assessment for Insulin Resistance. Among laboratory-based indices, the triglyceride–glucose index and the homeostasis model assessment showed good discriminative performance. Within lipid–anthropometry combinations, the triglyceride–glucose waist-to-height ratio and triglyceride–glucose waist circumference provided the best performance, each yielding an area under the curve of approximately 0.79. Several non-invasive indices also demonstrated good comparative performance. The bioimpedance-based somatotype adiposity index showed the highest overall performance among individual indices, with an area under the curve of 0.82, sensitivity of 78 percent, specificity of 80 percent, and an adjusted odds ratio of 14.9. These findings suggest that selected non-invasive anthropometric and body-composition indices may provide a practical and low-cost approach for assessing metabolic alterations associated with insulin resistance across glycemic groups, although further validation is required.

## Introduction

Insulin resistance (IR) is a metabolic abnormality characterized by a reduced biological response of peripheral tissues to insulin stimulation, resulting in impaired glucose disposal and compensatory hyperinsulinemia [1]. IR plays a central role in the pathogenesis of type 2 diabetes mellitus (T2DM), metabolic syndrome, and related cardiometabolic disorders [2]. Emerging evidence also links IR and prediabetes with broader clinical consequences, including cognitive impairment and cardiovascular dysfunction [3,4]. Individuals with prediabetes, representing an intermediate stage between normal glucose regulation and T2DM, frequently exhibit metabolic alterations associated with IR [5].

Early identification of metabolic alterations associated with insulin resistance may facilitate timely interventions aimed at reducing progression toward T2DM. The underlying mechanisms of prediabetes, such as impaired insulin signaling, adipocyte inflammation, and altered hepatic glucose output, mirror those in overt diabetes but differ in magnitude rather than nature [6,7].

The diagnosis of IR is primarily based on the quantification of tissue responsiveness to insulin and the efficiency of glucose utilization. The hyperinsulinemic-euglycemic clamp (HEC) remains the gold standard for assessing insulin sensitivity [8], but its complexity limits its clinical applicability. However, several surrogate markers have been developed using fasting biochemical parameters, such as the Homeostasis Model Assessment for IR (HOMA-IR) [9], Quantitative Insulin Sensitivity Check Index (QUICKI) [10], McAuley index [11], and the Triglyceride-Glucose (TyG) index [12], which are widely used in research and clinical practice. Modified indices combining biochemical and anthropometric data, such as TyG-Body Mass Index (BMI), TyG-Waist Circumference (WC), and TyG-Waist-to-Hip Ratio (WHR), have demonstrated improved discriminative performance and stronger associations with insulin resistance and metabolic risk in previous studies [13–15]. In addition, indices such as the Lipid Accumulation Product (LAP) and Visceral Adiposity Index (VAI) incorporate markers of central adiposity and lipid levels, providing a more integrative view of metabolic dysfunction [16,17].

However, these indices require blood sampling, limiting feasibility for large-scale or resource-limited screenings. In contrast, non-invasive measures based solely on anthropometry (BMI, WHR, Waist-to-Height Ratio (WHtR), Conicity Index (CI), Body Adiposity Index (BAI), and A Body Shape Index (ABSI)) and a few body composition parameters (Body fat % (BF%), Extracellular water %, and Dry Lean Weight (DLW%)) offer practical and low-cost alternatives for characterizing metabolic alterations and body composition patterns associated with metabolic dysfunction [18,19]. However, their comparative performance and relationship with IR-related metabolic alterations across glycemic groups remain underexplored. Because insulin resistance-related indices differ substantially in physiological basis, invasiveness, and feasibility for large-scale application [9–17], comparative evaluation within a single population may help identify indices with potential biological and practical relevance across glycemic groups.

**Fig 1:**
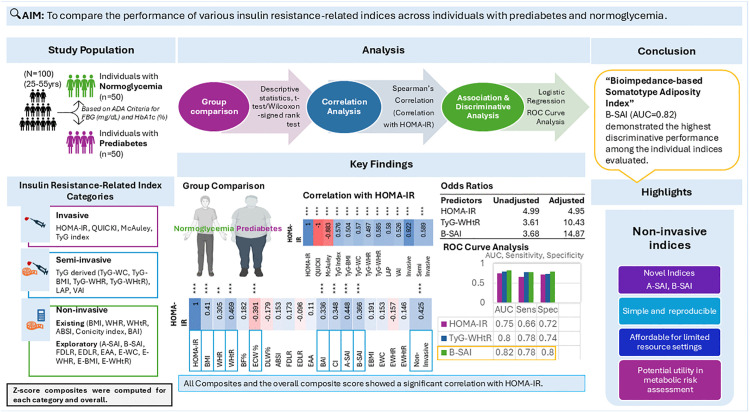
Graphical abstract.

Therefore, the present study was designed to evaluate a comprehensive panel of twenty-five candidate indices spanning invasive (Blood-based), semi-invasive (lipid and anthropometry-based), and non-invasive (anthropometric and body-composition-related) measures across individuals with prediabetes and normoglycemia. Composite z-scores were computed using indices demonstrating significant correlations with HOMA-IR within each category and as an overall composite score. HOMA-IR was used as a biological anchor for correlation analysis because it is a widely used surrogate marker of insulin resistance, allowing exploratory assessment of whether candidate indices may reflect metabolic alterations associated with insulin resistance. The objectives of the study were: (i) to compare differences in candidate indices across glycemic groups; (ii) to examine their relationships with HOMA-IR through correlation analysis; (iii) to evaluate the association between IR-related indices and prediabetes using logistic regression; and (iv) to assess the discriminative ability of IR-related indices in distinguishing prediabetes from normoglycemia using Receiver Operating Characteristic (ROC) curves. By integrating both traditional and exploratory measures, this study aimed to identify feasible and low-cost non-invasive indices with potential utility in characterizing metabolic alterations associated with IR across glycemic groups. A graphical overview of the study design, analytical framework, and key findings is presented in Fig 1.

## Materials and methods

### Study design

This was a cross-sectional sub-study based on the screening phase of a clinical trial conducted at Manipal Academy of Higher Education, Manipal. The study protocol was approved by the Institutional Ethics Committee of Kasturba Hospital (IEC: 723/2021), and the trial was prospectively registered with the Clinical Trials Registry - India (CTRI) (Registration number: CTRI/2022/04/042307, Registration date: 29 April 2022). All procedures were conducted in accordance with the principles of the Declaration of Helsinki, and all participants provided written informed consent.

Recruitment for the trial screening took place from June 14, 2022, to February 22, 2025.

### Participants

During the screening, South Asian (Indian) individuals were evaluated for glycemic status based on fasting blood glucose and HbA1c levels according to the American Diabetes Association (ADA) criteria [1]. The 50 individuals diagnosed with normoglycemia (fasting glucose <100 mg/dL and HbA1c < 5.7%) and the 50 individuals diagnosed with prediabetes (fasting glucose between 100–125 mg/dL and HbA1c between 5.7–6.4%) were included. Participants aged 25–55 years of either sex were eligible for inclusion if they met the criteria described above. Individuals with known diabetes mellitus, those who were pregnant or lactating, or those using medications affecting glucose or lipid metabolism were excluded. Participants with a history of chronic conditions such as hepatic, renal, endocrine, or cardiovascular disorders were also excluded.

### Participant characteristics

Demographic data, including age and gender, were recorded.

### Anthropometric assessment

Anthropometric measurements, including Height (cm), Weight (kg), WC, and hip circumference (HC), were obtained using standard protocols.

### Body composition analysis

Body composition parameters, including Body Fat percentage (BF%), Extracellular Water percentage (ECW%), and Dry Lean Weight percentage (DLW%), were assessed using the bioelectrical impedance analysis (BIA) method (Bodystat1500 MDD Model). The device operates at a fixed frequency of 50 kHz using a low-level alternating current (~200–400 μA) and employs a hand-to-foot tetrapolar technique with participants in the supine position. BIA is a validated and widely used method for body composition assessment in both clinical and research settings [18,20,21].

To ensure measurement reliability, standardized protocols were followed, including assessment in a fasting state, controlled hydration status, and appropriate electrode placement. Participants were positioned supine with limbs slightly apart, and measurements were taken after a stabilization period. The device was used in accordance with manufacturer guidelines. According to manufacturer specifications, the device measures impedance over a defined range with high precision (±2 Ω), supporting reproducible estimates of body composition. However, as with all BIA methods, measurements may be influenced by factors such as hydration status, recent physical activity, and electrode placement [22].

### Biochemical analysis

Fasting venous blood samples were collected after a minimum of 8 hours of overnight fasting to analyze Fasting Blood Glucose (FBG) (mg/dL), Fasting Insulin (μU/mL), Triglycerides (TG) (mg/dL), and High-Density Lipoprotein Cholesterol (HDL-C) (mg/dL). All the parameters were analyzed using standard enzymatic methods.

### Calculation of candidate indices

The study assessed twenty-five candidate indices classified as invasive, semi-invasive, and non-invasive, along with several exploratory hydration-adjusted indices.

### Invasive (blood-based) indices

Included established biochemical surrogates of IR, HOMA-IR [9], QUICKI [10], McAuley Index [11], and the TyG Index [12], calculated according to their original published equations

### Semi-invasive (lipid and anthropometric-based) indices

Indices incorporating biochemical and anthropometric measures were computed, including TyG-derived variants (TyG-BMI, TyG-WC, TyG-WHR, TyG-WHtR) [14,15,23], the LAP, and the VAI [16,17], following previously validated formulas.

### Non-invasive (anthropometric and bioimpedance-based) indices

Non-invasive markers derived solely from anthropometry and bioimpedance analysis were used to represent adiposity and somatic composition, including BMI, WHR, WHtR, CI, BAI, and A Body Shape Index (ABSI), computed per established definitions [24,25].

In addition to these established parameters, several exploratory indices were formulated to examine relationships between anthropometry, body composition, and hydration status.

Anthropometric Somatotype Adiposity Index (A-SAI) = BMI × WHtR

Bioimpedance Somatotype Adiposity Index (B-SAI) = (Body Fat % × WHtR) / ECW%

Fat-to-Dry Lean Ratio (FDLR) = Body Fat% / DLW%

ECW-to-Dry Lean Ratio (EDLR) = ECW % / DLW %

ECW-to-Adjusted Adiposity (EAA) = (BF % × ECW %) / DLW %

ECW-Adjusted WC (EWC) = ECW % × WC (cm)

ECW-Adjusted BMI (EBMI) = ECW % × BMI

ECW-Adjusted WHR (EWHR) = ECW % × WHR

ECW-Adjusted WHtR (EWHtR) = ECW % × WHtR

### Composite scores

Standardized z-scores were calculated for all indices to allow comparison across different measurement scales. Composite indices were generated by averaging z-scores within each category. Only indices demonstrating significant correlations with the Homeostasis Model Assessment for Insulin Resistance were included in composite score construction.

Invasive Composite Score: HOMA-IR, QUICKI, McAuley, TyG

Semi-Invasive Composite Score: TyG-derived indices (TyG-BMI, TyG-WC, TyG-WHR, TyG-WHtR) and LAP, and VAI.

Non-Invasive Composite Score: Anthropometric and body composition-based indices (BMI, WHR, WHtR, Conicity, BAI, A-SAI, B-SAI)

Overall Composite Score: Combined z-scores of all above indices

These composites provided dimensionless, standardized representations of combined index profiles for comparative evaluation across glycemic groups..

### Statistical analysis

Statistical analyses were conducted using Jamovi (version 2.5.3) and EZR (version 1.54). Continuous variables were presented as mean ± standard deviation (SD) or median (interquartile range [IQR]) according to distribution, while categorical variables were expressed as frequencies and percentages and compared using the Chi-square test. Normality of data was verified using the Shapiro-Wilk test. Between-group comparisons were performed using the independent t-test for normally distributed variables and the Mann-Whitney U test for non-normally distributed data.

Effect sizes were calculated using Cohen’s d for parametric variables, rank-biserial correlation (r) for non-parametric variables, and Cramér’s V for categorical variables to estimate the magnitude of group differences. Z-scores were computed for indices included in composite score construction to enable standardized comparisons and the generation of composite scores.

Relationships between HOMA-IR and candidate indices (invasive, semi-invasive, and non-invasive) were assessed using Spearman’s rank correlation coefficient (ρ) due to the non-normal distribution of biochemical parameters. Correlation analysis was performed to identify indices demonstrating relationships with IR-related metabolic alterations. Correlation strength was classified as very weak (< 0.20), weak (0.20–0.39), moderate (0.40–0.59), strong (0.60–0.79), or very strong (≥ 0.80). Heatmaps were generated to visualize the correlation patterns between indices using GraphPad Prism (Version: 8.5).

Indices demonstrating significant correlations with the HOMA-IR were included in subsequent logistic regression and ROC curve analyses. Prediabetes status served as the outcome for these analyses.

Binary logistic regression was performed to assess the association between IR-related indices and prediabetes status. Both unadjusted (Model 1) and multivariable-adjusted (Model 2) models were computed. Model 2 was adjusted for age, sex, and BMI, except where BMI exhibited multicollinearity (variance inflation factor > 5).

ROC analysis was performed to evaluate the discriminative ability of IR-related indices across glycemic groups and determine the Area Under the Curve (AUC), optimal cut-off values (Youden index), sensitivity, and specificity for each significant index. AUC values were interpreted as follows: 0.5–0.69 = poor, 0.7–0.79 = acceptable, 0.8–0.89 = good, and ≥ 0.9 = excellent discrimination.

All statistical tests were two-tailed, and a p-value < 0.05 was considered statistically significant.

## Results

### Participant characteristics and candidate indices

A total of 100 individuals were analyzed, including 50 with normoglycemia and 50 with prediabetes. Table 1 summarizes their demographic, anthropometric, body-composition, and biochemical characteristics. Individuals with prediabetes were significantly older than those with normoglycemia (45.30 ± 6.57 vs 39.60 ± 8.15 years; p < 0.001; d = 0.77). They also showed higher body weight (68.80 [58.20–81.20] vs 60.70 [55.20–72.90] kg; p = 0.020), waist circumference (90.30 ± 9.34 vs 83.20 ± 10.70 cm; p < 0.001), and hip circumference (99.00 [93.00–103.00] vs 94.50 [90.00–99.00] cm; p = 0.003), and a greater body-fat percentage (36.9 ± 6.0 vs 29.2 ± 7.8; p < 0.001; d = 1.11). Conversely, ECW% (21.00 ± 2.15 vs 23.60 ± 2.32; p < 0.001) and DLW% (16.30 ± 2.49 vs 19.80 ± 3.12; p < 0.001) were lower in individuals with prediabetes (d ≈ 1.10), indicating a reduced relative hydration and lean-mass proportion. Fasting glucose (112 [104–118] vs 89 [86–93] mg/dL; p < 0.001), insulin (12.70 [8.41–20.40] vs 8.89 [6.19–13.50] µIU/mL; p = 0.005), and triglyceride concentrations (132 [102–175] vs 92.50 [72–141] mg/dL; p < 0.001) were higher in prediabetes, whereas HDL-cholesterol tended to be lower (44.50 [39.00–51.00] vs 48.50 [40.50–63.80] mg/dL; p = 0.055).

**Table 1:** Demographic, anthropometric, body composition, and biochemical characteristics of study participants across prediabetes and normoglycemia.

Parameter	Normoglycemia (n = 50) (mean ± SD / median [IQR] / n [%])	Prediabetes (n = 50) (mean ± SD / median [IQR] / n [%])	p-value	Effect size
**Demographics**				
Age (years)	39.60 ± 8.15	45.30 ± 6.57	<0.001	−0.77
Gender (Male), n (%)	17 (34%)	19 (38%)	0.677	0.04
**Anthropometric Measures**				
Height (cm)	162 (157–170)	162 (158–169)	0.817	0.03
Weight (kg)	60.70 (55.20–72.90)	68.80 (58.20–81.20)	0.020	0.27
Waist Circumference (cm)	83.20 ± 10.70	90.30 ± 9.34	<0.001	−0.71
Hip Circumference (cm)	94.50 (90.00–99.00)	99.00 (93.00–103.00)	0.003	0.34
**Body Composition Measures**				
Body Fat %	29.20 ± 7.84	36.90 ± 5.97	<0.001	−1.11
ECW %	23.60 ± 2.32	21.00 ± 2.15	<0.001	1.17
DLW %	19.80 ± 3.12	16.30 ± 2.49	<0.001	1.10
**Key Markers**				
Fasting Glucose (mg/dL)	89 (86–93)	112 (104–118)	<0.001	1.00
Fasting Insulin (µIU/mL)	8.89 (6.19–13.50)	12.70 (8.41–20.40)	0.005	0.33
Triglycerides (mg/dL)	92.50 (72–141)	132 (102–175)	<0.001	0.38
HDL-C (mg/dL)	48.50 (40.50–63.80)	44.50 (39.00–51.00)	0.055	0.22

*Note.* Values are presented as mean ± standard deviation, median (interquartile range), or frequency (percentage), as appropriate. Comparisons between normoglycemia and prediabetes groups were performed using independent t-tests, Mann–Whitney U tests, or Chi-square tests, as applicable. p-values indicate group differences. Effect sizes (Cohen’s d, rank-biserial r or Cramer’s V) are reported to indicate the magnitude of differences.

Table 2 presents descriptive statistics for the invasive, semi-invasive, and non-invasive candidate indices. Among invasive indices, HOMA-IR values were higher in individuals with prediabetes (3.33 [2.21–5.62] vs 1.98 [1.38–2.94]; p < 0.001; d = 0.49), while QUICKI (0.32 ± 0.03 vs 0.35 ± 0.03; p < 0.001; d = 0.92) and McAuley (6.08 [5.19–6.85] vs 6.98 [5.97–8.75]; p < 0.001; d = 0.40) indices were lower. The TyG Index was also significantly elevated (8.90 ± 0.35 vs 8.40 ± 0.47; p < 0.001; d = 1.20).

**Table 2:** Descriptive statistics of invasive, semi-invasive, and non-invasive candidate indices across prediabetes and normoglycemia.

Index	Normoglycemia (n = 50) (mean ± SD / median [IQR])	Prediabetes (n = 50) (mean ± SD / median [IQR])	p-value	Effect Size
**Invasive Indices**				
HOMA-IR	1.98 (1.38–2.94)	3.33 (2.21–5.62)	<0.001	0.49
QUICKI	0.35 ± 0.03	0.32 ± 0.03	<0.001	0.92
McAuley	6.98 (5.97–8.75)	6.08 (5.19–6.85)	<0.001	0.40
TyG Index	8.40 ± 0.47	8.90 ± 0.35	<0.001	−1.20
**Semi-Invasive Indices**				
TyG-BMI	197 ± 35.90	231 ± 35.80	<0.001	−0.93
TyG-WC	700 ± 105	804 ± 94.70	<0.001	−1.05
TyG-WHR	7.36 ± 0.78	8.13 ± 0.66	<0.001	−1.07
TyG-WHtR	4.27 ± 0.63	4.90 ± 0.46	<0.001	−1.14
LAP	22.80 (15.90–36.10)	44.50 (31.90–61.70)	<0.001	0.54
VAI	1.31 (0.99–2.40)	2.09 (1.43–2.95)	0.001	0.38
**Non-invasive Indices**				
BMI(kg/m²)	23.50 ± 3.85	25.90 ± 3.81	0.002	−0.64
WHR	0.88 ± 0.07	0.91 ± 0.06	0.004	−0.58
WHtR	0.49 (0.46–0.55)	0.55 (0.52–0.57)	<0.001	0.47
ABSI	0.080 ± 0.005	0.081 ± 0.004	0.135	−0.30
FDLR	1.57 ± 0.68	2.29 ± 0.60	<0.001	−1.13
EDLR	1.23 (1.15–1.31)	1.23 (1.21–1.27)	0.785	0.03
EAA	36.20 ± 13.10	47.50 ± 10.80	<0.001	−0.94
A-SAI	11.60 (9.54–13.50)	14.20 (12.60–16.50)	<0.001	0.42
B-SAI	0.61 (0.47–0.75)	1.01 (0.80–1.11)	<0.001	0.64
EWC	1950 ± 197	1892 ± 241	0.194	0.26
EBMI	548 ± 63.20	541 ± 80.40	0.642	0.09
EWHR	20.40 (19.30–21.80)	18.80 (17.30–21.30)	0.002	0.35
EWHtR	11.90 ± 0.97	11.50 ± 1.13	0.086	0.35
Conicity Index	1.23 ± 0.08	1.27 ± 0.06	0.004	−0.59
BAI	26.50 (24.50–30.30)	28.20 (26.40–31.90)	0.023	0.26
**Composite Scores**				
Invasive CompScore (z-score composite)	−0.41 ± 0.74	0.41 ± 0.83	<0.001	−1.05
Semi-Invasive CompScore (z-score composite)	−0.43 ± 0.81	0.43 ± 0.72	<0.001	−1.11
Non-invasive CompScore (z-score composite)	−0.570 (−0.901 – −0.003)	0.365 (−0.071–0.659)	<0.001	0.59
Overall CompScore_0_100	−0.40 ± 0.67	0.40 ± 0.55	<0.001	−1.29

*Note.* Comparison of candidate indices and composite z-scores between individuals with normoglycemia and prediabetes. Values are expressed as mean ± SD or median (interquartile range). p-values represent between-group differences. Effect sizes (Cohen’s d or rank-biserial r) are reported to indicate the magnitude of differences.

Within semi-invasive indices, all TyG-derived measures were significantly higher in the prediabetes group, including TyG-BMI (231.00 ± 35.80 vs 197.00 ± 35.90), TyG-WC (804.00 ± 94.70 vs 700.00 ± 105.00), TyG-WHR (8.13 ± 0.66 vs 7.36 ± 0.78), and TyG-WHtR (4.90 ± 0.46 vs 4.27 ± 0.63) (all p < 0.001) with large standardized mean differences (|d| = 0.93–1.14). Lipid-based indices showed similar trends: LAP (44.50 [31.90–61.70] vs 22.80 [15.90–36.10]; p < 0.001; d = 0.54) and VAI (2.09 [1.43–2.95] vs 1.31 [0.99–2.40]; p = 0.001; d = 0.38) were elevated in individuals with prediabetes.

For non-invasive indices, BMI (25.90 ± 3.81 vs 23.50 ± 3.85; p = 0.002; d = 0.64), WHR (0.91 ± 0.06 vs 0.88 ± 0.07; p = 0.004; d = 0.58), WHtR (0.55 [0.52–0.57] vs 0.49 [0.46–0.55]; p < 0.001; d = 0.47), Conicity Index (1.27 ± 0.06 vs 1.23 ± 0.08; p = 0.004; d = 0.59), and BAI (28.20 [26.40–31.90] vs 26.50 [24.50–30.30]; p = 0.023; d = 0.26) were significantly higher in individuals with prediabetes, whereas ABSI did not differ (0.081 ± 0.004 vs 0.080 ± 0.005; p = 0.135). Among body-composition-based ratios, FDLR (2.29 ± 0.60 vs 1.57 ± 0.68; p < 0.001; d = 1.13) and EAA (47.50 ± 10.80 vs 36.20 ± 13.10; p < 0.001; d = 0.94) were higher, while EDLR showed no difference (1.23 [1.21–1.27] vs 1.23 [1.15–1.31]; p = 0.785). Both Somatotype Adiposity Indices, A-SAI (14.20 [12.60–16.50] vs 11.60 [9.54–13.50]) and B-SAI (1.01 [0.80–1.11] vs 0.61 [0.47–0.75]), were elevated in individuals with prediabetes (both p < 0.001; d = 0.42 and 0.64, respectively). Among the ECW-adjusted exploratory ratios, EWHR was significantly lower in the individuals with prediabetes (18.80 [17.30–21.30] vs 20.40 [19.30–21.80]; p = 0.002; d = 0.35), whereas the remaining indices (EWC: 1892 ± 241 vs 1950 ± 197; p = 0.194; EBMI: 541 ± 80.40 vs 548 ± 63.20; p = 0.642; EWHtR: 11.50 ± 1.13 vs 11.90 ± 0.97; p = 0.086) did not differ significantly.

All composite z-score-based indices (invasive, semi-invasive, and non-invasive) were significantly higher in prediabetes (invasive: 0.41 ± 0.83 vs − 0.41 ± 0.74; semi-invasive: 0.43 ± 0.72 vs −0.43 ± 0.81; non-invasive: 0.365 [−0.071–0.659] vs − 0.570 [−0.901 – −0.003]; all p < 0.001) when compared to the normoglycemic group, with moderate-to-large standardized mean differences (|d| ≈ 0.59–1.11). The overall Composite Score showed the greatest separation between groups (0.40 ± 0.55 vs −0.40 ± 0.67; p < 0.001; d = 1.29). Overall, medium-to-large effect sizes across most parameters between individuals with normoglycemia and prediabetes.

### Correlation analysis

Spearman’s correlation analysis was conducted to examine the relationships between candidate indices and HOMA-IR. Seventeen of the twenty-five candidate indices demonstrated significant correlations with HOMA-IR and were subsequently included in logistic regression and receiver operating characteristic analyses.

Among invasive and semi-invasive indices, QUICKI correlated very strongly and inversely with HOMA-IR (ρ = −1.00, p < 0.001), and the McAuley index also showed a very strong inverse relationship (ρ = −0.883, p < 0.001). The Triglyceride-Glucose (TyG) index showed a moderate positive correlation with HOMA-IR (ρ = 0.576, p < 0.001). Its derived measures were similarly correlated with HOMA-IR: TyG-BMI (ρ = 0.504, p < 0.001), TyG-WC (ρ = 0.570, p < 0.001), TyG-WHR (ρ = 0.497, p < 0.001), and TyG-WHtR (ρ = 0.585, p < 0.001), all demonstrating moderate positive correlations. Lipid-based indices also showed moderate positive correlations with HOMA-IR (LAP ρ = 0.580, p < 0.001; VAI ρ = 0.526, p < 0.001). The invasive composite score correlated very strongly with HOMA-IR (ρ = 0.922, p < 0.001), and the semi-invasive composite showed a moderate positive correlation with HOMA-IR (ρ = 0.589, p < 0.001).

Within non-invasive indices, BMI (ρ = 0.410, p < 0.001) and WHtR (ρ = 0.469, p < 0.001) showed moderate positive correlations, while WHR (ρ = 0.305, p = 0.002) showed a weak positive correlation with HOMA-IR. Body-fat % (ρ = 0.182) and DLW % (ρ = −0.179) showed very weak, non-significant correlations with HOMA-IR (p > 0.05). ECW% demonstrated a weak inverse correlation (ρ = −0.391, p < 0.001) with HOMA-IR. Among additional non-invasive indices, BAI (ρ = 0.336, p < 0.001) and Conicity Index (ρ = 0.348, p < 0.001) demonstrated weak correlations with HOMA-IR, while ABSI (ρ = 0.153, p = 0.127) was not significantly correlated with HOMA-IR. Of the composition-based ratios, FDLR (ρ = 0.173, p = 0.086), EDLR (ρ = −0.096, p = 0.341), and EAA (ρ = 0.110, p = 0.277) showed very weak, non-significant relationships with HOMA-IR. Both Somatotype Adiposity Indices, A-SAI (ρ = 0.448, p < 0.001) and B-SAI (ρ = 0.366, p < 0.001), showed moderate and weak positive correlations with HOMA-IR, respectively. Other ECW-adjusted ratios (EBMI, EWC, EWHR, EWHtR) were not significantly correlated with HOMA-IR (p > 0.05). The non-invasive composite score showed a moderate positive correlation with HOMA-IR (ρ = 0.425, p < 0.001).

**Fig 2:**
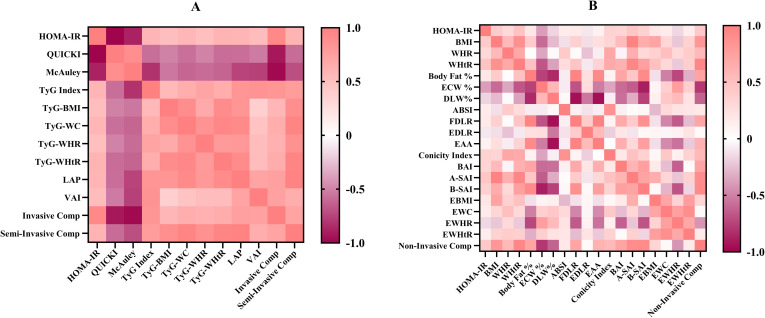
Heatmap of Spearman’s correlation coefficients (ρ) between HOMA-IR and candidate indices. **(A)** Invasive and semi-invasive indices; **(B)** Non-invasive and body composition-based indices. Darker shades represent stronger positive correlations, while lighter or neutral colors indicate weaker or inverse relationships.

These associations are visualized in **Fig 2**, which presents heatmaps of correlation strength for (A) invasive and semi-invasive indices and (B) non-invasive indices. The heatmaps visually summarize the correlation strengths across invasive, semi-invasive, and non-invasive indices. A detailed correlation matrix for all variables is included in the **S1 File**, supporting the summary results presented here.

### Logistic regression analysis

Binary logistic regression was performed to assess the association between IR-related indices and prediabetes status. Multivariable-adjusted model (Model 2) included age, sex, and BMI. Table 3 summarizes the unadjusted and adjusted odds ratios (ORs) with 95% confidence intervals.

**Table 3:** Binary logistic regression analysis of the association between insulin resistance-related indices and prediabetes status.

Predictors (per 1-SD increase)	Model 1: Unadjusted OR (95% CI)	p-value	Model 2: Adjusted OR (95% CI)	p-value
**Invasive Indices**				
HOMA-IR	4.99 (2.00-12.48)	**<0.001**	4.95 (1.78-13.77)	**0.002**
QUICKI (Inverse)	2.79 (1.66-4.70)	**<0.001**	2.93 (1.60-5.37)	**<0.001**
McAuley (Inverse)	2.24 (1.38-3.63)	**0.001**	2.58 (1.42-4.66)	**0.002**
TyG Index	3.88 (2.16-6.97)	**<0.001**	5.68 (2.62-12.28)	**<0.001**
**Semi-Invasive Indices**				
TyG-BMI	2.74 (1.66-4.54)	**<0.001**	3.05 (1.73-5.36)	**<0.001**
TyG-WC	3.20 (1.87-5.48)	**<0.001**	11.07 (3.41-35.89)	**<0.001**
TyG-WHR	3.30 (1.91-5.71)	**<0.001**	6.25 (2.67-14.64)	**<0.001**
TyG-WHtR	3.61 (2.06-6.35)	**<0.001**	10.43 (3.29-33.06)	**<0.001**
LAP	2.83 (1.64-4.90)	**<0.001**	3.29 (1.53-7.10)	**0.002**
VAI	2.03 (1.23-3.35)	**0.005**	2.55 (1.37-4.73)	**0.003**
**Non-Invasive Indices**				
BMI	1.96 (1.25-3.07)	**0.003**	1.99 (1.22-3.26)	**0.006**
WHR	1.85 (1.19-2.88)	**0.007**	1.84 (0.97-3.48)	**0.062**
WHtR	2.27 (1.42-3.64)	**<0.001**	2.37 (0.92-6.08)	**0.073**
A-SAI	2.05 (1.30-3.23)	**0.002**	2.07 (1.26-3.41)	**0.004**
B-SAI	3.68 (2.07-6.57)	**<0.001**	14.87 (4.28-51.62)	**<0.001**
Conicity Index	1.89 (1.20-2.99)	**0.006**	1.78 (1.04-3.04)	**0.036**
BAI	1.58 (1.03-2.43)	**0.037**	1.15 (0.56-2.38)	**0.700**
**Composite Scores**				
Invasive composite score	4.20 (2.12-8.30)	**<0.001**	5.17 (2.30-11.60)	**<0.001**
Semi-invasive composite score	4.20 (2.21-7.97)	**<0.001**	10.77 (3.26-35.61)	**<0.001**
Non-invasive composite score	4.96 (2.37-10.37)	**<0.001**	19.26 (3.64-101.92)	**<0.001**
Overall composite	8.37 (3.45-20.31)	**<0.001**	33.99 (6.92-167.08)	**<0.001**

*Note.* Unadjusted (Model 1) and adjusted (Model 2) odds ratios (ORs) with 95% confidence intervals (CIs) for each standardized (z-score) index. Model 2 was adjusted for age, sex, and BMI, except where BMI was excluded due to multicollinearity (variance inflation factor > 5). Statistical significance was determined at *p* < 0.05. A higher OR indicates greater odds of prediabetes per 1 SD increase in the respective index.

Among invasive indices, each one-standard-deviation increase in HOMA-IR was associated with nearly a fivefold higher odds of prediabetes (adjusted OR = 4.95, 95% CI: 1.78–13.77, p = 0.002). QUICKI and McAuley indices, which are inversely related to IR, also demonstrated significant associations (adjusted OR = 2.93 and 2.58, respectively; both p ≤ 0.002). The TyG Index showed strong association with prediabetes (adjusted OR = 5.68, 95% CI: 2.62–12.28, p < 0.001). The invasive composite score also demonstrated a significant association with prediabetes (adjusted OR = 5.17, 95% CI: 2.30–11.60, p < 0.001).

Among semi-invasive indices, TyG derivatives demonstrated significant associations with prediabetes. TyG-WHtR (adjusted OR = 10.43, 95% CI: 3.29–33.06, p < 0.001) and TyG-WC (adjusted OR = 11.07, 95% CI: 3.41–35.89, p < 0.001) were associated with higher odds of prediabetes, followed by TyG-WHR (adjusted OR = 6.25, 95% CI: 2.67–14.64, p < 0.001) and TyG-BMI (adjusted OR = 3.05, p < 0.001). Both LAP and VAI also showed significant associations with prediabetes (adjusted OR = 3.29 and 2.55, respectively). The semi-invasive composite showed a tenfold higher odds of prediabetes (adjusted OR = 10.77, 95% CI: 3.26–35.61, p < 0.001).

Within non-invasive indices, BMI, WHR, WHtR, Conicity Index, A-SAI, and B-SAI all showed significant unadjusted associations with prediabetes (p < 0.01). After adjustment, BMI (adjusted OR = 1.99, 95% CI: 1.22–3.26, p = 0.006), A-SAI (adjusted OR = 2.07, 95% CI: 1.26–3.41, p = 0.004), and B-SAI (adjusted OR = 14.87, 95% CI: 4.28–51.62, p < 0.001) remained independently associated with prediabetes. Conicity Index also retained significance (adjusted OR = 1.78, p = 0.036), while WHtR and BAI lost statistical significance after adjustment for age, sex, and BMI. The non-invasive composite score demonstrated nearly a twentyfold higher odds of prediabetes (adjusted OR = 19.26, 95% CI: 3.64–101.92, p < 0.001).

Overall, the overall composite score showed a significant association with prediabetes, with an adjusted odds ratio of 33.99 (95% CI: 6.92–167.08, p < 0.001).

### Receiver operating characteristic curve analysis

ROC curve analysis was performed to evaluate the discriminative ability of each IR-related index and composite score in distinguishing individuals with prediabetes from those with normoglycemia (Table 4).

**Table 4:** Receiver Operating Characteristic (ROC) analysis of insulin resistance-related indices for discriminating individuals with prediabetes from Normoglycemia.

Marker (per SD)	AUC (95% CI)	Optimal Cut-off	Sensitivity (%)	Specificity (%)
**Invasive Indices**				
HOMA IR	0.75	2.86	66	72
QUICKI	0.75	0.33	66	72
McAuley	0.70	6.27	60	70
TyG Index	0.79	8.37	98	56
**Semi-Invasive Indices**				
TyG-BMI	0.75	210.90	76	72
TyG-WC	0.77	714.20	86	64
TyG-WHR	0.78	7.41	86	56
TyG-WHtR	0.80	4.64	78	74
LAP	0.77	36.62	70	80
VAI	0.69	1.32	80	52
**Non-Invasive Indices**				
BMI	0.69	22.41	88	44
WHR	0.67	0.89	70	70
WHtR	0.74	0.50	88	58
A-SAI	0.71	12.74	74	68
B-SAI	0.82	0.79	78	80
Conicity Index	0.68	1.24	80	58
BAI	0.63	26.66	72	52
**Composite Scores**				
Invasive composite	0.77	0.22	60	84
Semi-invasive composite	0.79	−0.03	78	72
Non-invasive composite	0.80	−0.36	98	58
Overall Composite	0.82	−0.08	88	72

*Note.* AUC, sensitivity, specificity, and optimal cut-off values (Youden index) for invasive, semi-invasive, and non-invasive indices and composite scores. Higher AUC values indicate greater discriminative ability.

Among invasive indices, HOMA-IR and QUICKI both demonstrated good discriminative ability (AUC = 0.75 for each; sensitivity 66%, specificity 72%). The McAuley Index showed slightly lower performance (AUC = 0.70). The TyG Index, another blood-based marker incorporating fasting triglyceride and glucose levels, also exhibited good discriminative ability (AUC = 0.79; cut-off 8.37; sensitivity 98%, specificity 56%), with performance comparable to HOMA-IR.

Within semi-invasive indices, the TyG-derived parameters displayed consistent and high discriminative capacity. TyG-WHtR (AUC = 0.80), TyG-WHR (AUC = 0.78), TyG-WC (AUC = 0.77), and TyG-BMI (AUC = 0.75) all demonstrated good discriminative ability across prediabetes and normoglycemia. Lipid-based indices also performed well: LAP (AUC = 0.77; sensitivity 70%, specificity 80%) and VAI (AUC = 0.69) exhibited moderate discriminative ability. The semi-invasive composite score achieved an AUC of 0.79 (sensitivity 78%, specificity 72%).

Among non-invasive indices, B-SAI demonstrated the highest discriminative ability (AUC = 0.82; sensitivity 78%, specificity 80%), exceeding that of BMI (AUC = 0.69), Conicity Index (AUC = 0.68), and A-SAI (AUC = 0.71). WHtR also showed fair discriminative ability (AUC = 0.74; sensitivity 88%, specificity 58%). The non-invasive composite achieved an AUC of 0.80 (sensitivity 98%, specificity 58%).

Across all parameters, the overall composite index yielded the highest AUC (0.82; sensitivity, 88%; specificity, 72%), demonstrating good discrimination between individuals with prediabetes and normoglycemia.

### Summary of key findings

Both biochemical and non-invasive indices demonstrated strong discrimination between individuals with prediabetes and normoglycemia. Among invasive measures, HOMA-IR and TyG Index showed the highest discriminative ability with balanced sensitivity and specificity. Semi-invasive indices, particularly TyG-WHtR and TyG-WC, demonstrated good discriminative ability. Notably, several non-invasive indices also showed discriminative performance comparable to biochemical markers. B-SAI demonstrated the highest AUC (0.82) among individual IR-related indices. These findings indicate that simple anthropometric and bioimpedance-derived indices may have potential utility in characterizing metabolic alterations associated with insulin resistance across prediabetes and normoglycemia.

## Discussion

### Overview of the study

This study compared invasive, semi-invasive, and non-invasive insulin resistance (IR)-related indices in individuals with prediabetes and in those with normoglycemia. Prediabetes was associated with higher adiposity and elevated IR-related markers, consistent with early metabolic alteration. While the Homeostasis Model Assessment for IR (HOMA-IR) (AUC = 0.75) and Triglyceride-Glucose index (TyG index) (AUC = 0.79) demonstrated good discriminative ability across glycemic groups, the novel indices Anthropometric Somatotype Adiposity Index (A-SAI) (AUC = 0.71) and Bioimpedance Somatotype Adiposity Index (B-SAI) (AUC = 0.82) also showed comparable performance in distinguishing prediabetes from normoglycemia. These findings support the potential utility of selected non-invasive indices in characterizing metabolic alterations associated with IR across prediabetes and normoglycemia.

### Comparison with previous research

The performance of HOMA-IR and the TyG index observed here is consistent with previous findings identifying both as surrogate markers of IR and metabolic risk across populations [12,26]. TyG-derived parameters (Triglyceride-Glucose Body Mass Index [TyG-BMI] (AUC = 0.75), Triglyceride-Glucose Waist Circumference [TyG-WC] (AUC = 0.77), Triglyceride-Glucose Waist-to-Hip Ratio [TyG-WHR] (AUC = 0.78), Triglyceride-Glucose Waist-to-Height Ratio [TyG-WHtR] (AUC = 0.80)) in our analysis also demonstrated comparable Areas under the curves (AUCs), consistent with previous studies evaluating these indices against clamp-based and biochemical measures and in relation to prediabetes risk [12–15,23,26,27]. By combining adiposity and lipid components, TyG-derived indices may reflect metabolic alterations associated with dyslipidemia and glucose dysregulation. Lipid-based indices, Lipid Accumulation Product (LAP) (AUC = 0.77) and Visceral Adiposity Index (VAI) (AUC = 0.69), demonstrated moderate discriminative ability, consistent with evidence linking visceral lipid accumulation and triglyceride metabolism with metabolic alterations associated with IR [16,17].

The most notable observation in our study is that the non-invasive indices B-SAI and A-SAI achieved AUCs of 0.82 and 0.71, showing performance comparable to invasive biochemical markers such as HOMA-IR and TyG index in distinguishing prediabetes from normoglycemia. These findings align with recent work showing the relevance of body composition-based phenotyping [19], suggesting that indices integrating anthropometric and body composition parameters may improve discrimination of metabolic phenotypes compared with individual anthropometric measures such as BMI. A recent study on Young Adult men has evaluated simple anthropometry-based indices in relation to measures such as HOMA-IR, supporting the potential relevance of non-invasive predictors to metabolic alterations associated with IR [23]. In contrast, Extracellular Water (ECW) derived ratios were not significantly correlated with HOMA-IR, though ECW% alone exhibited a weak inverse relationship with HOMA-IR. This suggests that hydration or lean-tissue balance may reflect underlying inflammatory or vascular influences rather than insulin action directly.

An Indian study also reported that ECW% (as a percentage of body weight) modestly differentiated individuals with prediabetes (AUC ≈ 0.70) but primarily reflected adiposity and inflammatory burden rather than direct glucose dysregulation [18]. Taken together, these results indicate that integrating anthropometric and body-composition parameters may improve characterization of metabolic alterations across glycemic groups. Our data extend previous findings by demonstrating that these non-invasive indices retain comparable performance, particularly among individuals with prediabetes, where early metabolic-risk assessment may be clinically relevant.

### Clinical and physiological implications

From a clinical standpoint, the feasibility of using anthropometric- and bioimpedance-based indices, such as A-SAI, B-SAI, and Conicity Index, holds significant potential in community and low-resource settings where biochemical assays are limited. These indices are entirely non-invasive, reproducible, and low-cost, relying only on simple anthropometric or bioimpedance data. Their performance in distinguishing prediabetes from normoglycemia was comparable to that of HOMA-IR (AUC = 0.75) and TyG (AUC = 0.79) suggesting that alterations in anthropometry, adiposity, and extracellular water may be associated with metabolic dysfunction across glycemic groups. Such structural and compositional alterations have previously been associated with inflammatory, oxidative, and lipotoxic mechanisms [28–30]. Hence, these indices may reflect phenotypic patterns associated with metabolic stress and altered body composition across glycemic groups.

Although ECW%-based ratios were not significantly correlated with HOMA-IR, the weak correlation of ECW% with HOMA-IR suggests an indirect association between hydration, inflammation, and IR. Because ECW% primarily reflects fluid distribution, it was not classified as an IR-related index in this study. The distinction suggests that anthropometric and adiposity-based parameters showed stronger associations with HOMA-IR than hydration-related parameters alone.

Implementing non-invasive indices in primary-care or occupational health screening could facilitate earlier identification of individuals at increased metabolic risk and enable timely lifestyle or dietary interventions. Because they are reproducible, inexpensive, and population-adaptable, they could be introduced to large-scale metabolic-risk assessment and longitudinal follow-up programs, especially in regions where invasive testing is not routinely feasible.

### Strengths, novelty, and future directions

A key strength of this study is its comprehensive and structured evaluation of IR-related indices across biochemical, lipid-anthropometric, and fully non-invasive domains within the same well-characterized cohort. By applying uniform z-score standardization, the analysis allowed direct comparison of indices that differ widely in scale, physiological basis, and measurement modality, an approach rarely undertaken in IR research. The integration of multiple index categories, followed by correlation, regression, and discrimination analyses within a single analytic framework, offers a more complete physiological perspective on early metabolic dysfunction than studies that examine these markers in isolation. The development and evaluation of two somatotype-based indices (A-SAI and B-SAI) constitute a novel methodological contribution. These indices combine simple anthropometric and bioimpedance-derived parameters to capture body-shape patterns and fluid-fat distribution, yielding discriminative performance comparable to traditional biochemical measures. Importantly, this study extends the evidence base by testing these indices specifically in individuals with prediabetes, a population where subtle metabolic alterations may not be fully characterized using routine clinical markers alone. The side-by-side comparison of 25 candidate indices, including nine exploratory non-invasive measures, provides one of the most detailed assessments to date of which indices demonstrate practical applicability and discriminative utility. Collectively, these features position the study as a relevant contribution for advancing low-cost, scalable, and population-adaptable strategies for metabolic-risk assessment across glycemic groups.

Limitations include the cross-sectional design, which restricts causal inference and limits assessment of longitudinal performance over time, and the modest sample size, which precludes sex-specific or subgroup analyses and may limit generalizability. The study population consisted exclusively of South Asian (Indian) individuals; therefore, the findings and optimal cut-off values may not be directly generalizable to other ethnic groups with different adiposity and body-composition characteristics and may require population-specific recalibration. In addition, given the evaluation of multiple indices and composite scores within the same dataset, the possibility of overfitting cannot be excluded. Furthermore, physical activity, dietary intake, smoking status, alcohol intake, and socioeconomic factors were not included in the present analysis; adjustment was limited to age, sex, and BMI. Future studies should incorporate these variables for more comprehensive modeling. Formal statistical comparison of AUCs (e.g., DeLong test) was not performed, as the study was primarily designed to evaluate discriminative performance rather than to test differences between AUCs. Future studies should validate these indices longitudinally across diverse populations, explore population-specific cut-offs, and integrate them into multivariate prediction models combining clinical and lifestyle factors. Future studies should also evaluate the direct performance of these indices against established IR measures such as HOMA-IR thresholds or hyperinsulinemic-euglycemic clamp-derived assessments.

## Conclusion

In conclusion, invasive, semi-invasive, and non-invasive IR-related indices demonstrated varying degrees of association and discriminative ability across prediabetes and normoglycemia. Among the evaluated non-invasive measures, B-SAI showed the highest discriminative performance, while A-SAI also demonstrated meaningful associations with HOMA-IR and glycemic-group differences. These findings suggest that selected anthropometric and body-composition-based indices may have potential utility as low-cost and scalable tools for metabolic-risk assessment across glycemic groups. However, further longitudinal studies and direct validation against established IR measures are required before clinical application.

## Supporting information

S1 File Detailed correlation matrix for all variables. (XLSX)

S2 File Study dataset. (XLSX)
